# Diagnostic Potential of Low Serum Platelet, Albumin and Prolong PT-INR for Overactive Bladder and Nocturia in Chronic Hepatitis-Related Liver Cirrhosis

**DOI:** 10.3390/jcm10132838

**Published:** 2021-06-27

**Authors:** Po-Heng Chuang, Yi-Huei Chang, Po-Jen Hsiao, Eric Chieh-Lung Chou

**Affiliations:** 1 Center for Digestive Medicine, Department of Internal Medicine, China Medical University Hospital, Taichung City 40402, Taiwan; poheng2@yahoo.com.tw; 2 Department of Urology, China Medical University Hospital, China Medical University, Taichung City 40402, Taiwan; yihueichang1006@gmail.com

**Keywords:** overactive bladder, liver cirrhosis, nocturia, frequency, urgency, incontinence

## Abstract

Overactive bladder (OAB) is defined as urgency, usually with frequency, nocturia, and incontinence. Patients with liver cirrhosis often present with urinary complaints. The possible reason for this is fluid redistribution, which may induce OAB resulting from portal hypertension and ascites. We conducted this study to investigate predictors of OAB in cirrhotic patients. A total of 164 patients with chronic viral hepatitis-related liver cirrhosis were enrolled and 158 (96.3%) completed the Overactive Bladder Symptoms Score (OABSS) questionnaire. Age, severity of liver cirrhosis, comorbidities, serum sodium level, use of diuretics, body mass index and renal function were also recorded. In the study cohort, the prevalence of OAB was 31.01% and the prevalence of urge incontinence (OAB wet) was 18.3%. Patients with an urgency score ≥2 in OABSS had a significantly lower platelet level (*p* = 0.025) regardless of the use of diuretics. In addition, 98 patients (62%) with nocturia and 29 patients (18%) with urge incontinence had significantly lower levels of serum albumin (*p* = 0.028 and 0.044, respectively). In conclusion, patients with liver cirrhosis have a high prevalence of overactive bladder. A low platelet and low serum albumin level in these patients may be predictors for overactive bladder. And longer PT-INR is also a possible biomarker for nocturia.

## 1. Introduction

Over the past several decades, liver diseases and cirrhosis have risen to become one of the leading causes of death and illness worldwide. In 2017, cirrhosis caused more than 1.32 million deaths globally. The number of prevalent cases of decompensated and compensated cirrhosis globally increased to over 10.6 million and 112 million, respectively. In 2017, the age-standardized prevalence of decompensated cirrhosis and compensated cirrhosis reached 132.5 per 100,000 and 1395 per 100,000 population, respectively [1]. Liver cirrhosis is a late-stage progressive hepatic fibrosis and involves the formation of regenerative nodules. The clinical course of patients with advanced cirrhosis is often complicated by a number of important sequelae that can occur regardless of the underlying cause of the liver disease [2]. Symptoms of cirrhosis include anorexia, weight loss, weakness, fatigue, jaundice, itching, and signs of upper gastrointestinal bleeding (hematemesis, melena, bloody stool), and hepatic encephalopathy is commonly observed by hepatologists. Urinary tract infections are commonly observed in liver cirrhosis patients despite seemingly few report of such findings in the literature.

Overactive bladder (OAB) is a storage lower urinary tract symptom (LUTS), defined by the International Continence Society (ICS) as urgency with or without urge urinary in-continence, usually with frequency and nocturia in the absence of urinary tract infections or other obvious pathology [3]. The prevalence of OAB is currently around 10% globally, and it is expected to keep increasing [4,5]. The Epidemiology Urinary Incontinence and Comorbidities (EPIC) study [6] reported similar prevalence rates of OAB in men (10.8%) and women (12.8%), with an increasing prevalence with advanced age. The reported prevalence rates of OAB and urge incontinence in subjects aged between 60 and 69 years in the EPIC study were 18% and 2.5%, respectively. Studies have shown that OAB seems to be more prevalent in East Asian people. A multi-country study conducted in China, Taiwan and South Korea found that the overall prevalence of OAB in adults aged ≥40 years was 20.8% (22.1% for women, 19.5% for men) [7]. Yu et al. reported an age-adjusted prevalence rate of OAB in patients aged 60 to 69 years of 16.9% in a questionnaire survey from 1827 community dwelling adults in a Taiwan population [8]. OAB is associated with a poor quality of life, diurnal fatigue, decreased concentration, lower performance at work, and accidents because of cognitive and motor impairment [9]. Therefore, an increasing amount of research has focused on OAB and the resulting health consequences. Due to high prevalence of both OAB and liver cirrhosis among East Asians, we conducted an observational study to assess the association between liver cirrhosis and OAB and to explore the diagnostic potential liver function test parameters in chronic liver cirrhosis patients susceptible to LUTS.

## 2. Materials and Methods

This study enrolled 164 patients with chronic viral hepatitis-related liver cirrhosis who attended the outpatient hepatogastroenterology clinics at a tertiary referral medical center between March 2014 and June 2018. Patients with primarily neurogenic voiding dysfunction, stress urinary incontinence, refractory urinary tract infection, urolithiasis, proven interstitial cystitis/bladder pain syndrome, bladder or prostate malignancy, benign prostate enlargement or LUTS under medication control or prostatic surgery, and bladder outlet obstruction such as urethral stricture were precluded from enrollment. The Institutional Review Board and the Ethics Committee of the hospital (CMUH102-REC2-038) approved this study.

All patients received face-to-face consultation from the same urologist (Eric Chieh-Lung Chou) to discuss the outcomes of the self-administered questionnaire, the Over-active Bladder Symptoms Score (OABSS).The OABSS is a four-item questionnaire developed by Homma et al., and is used to assess the symptoms of OAB. The total OABSS score is the sum of scores from these four questions: daytime frequency (score 0–2), nighttime frequency (score 0–3), urgency (score 0–5), and urgency incontinence (score 0–5) [10]. Of the 164 patients, 158 (96.3%) completed the questionnaire, but six did not respond due to malaise, refusal, or dementia.

Age, history of diabetes mellitus, hypertension, and dyslipidemia, severity of liver cirrhosis (Child Pugh classification, including hypoalbuminemia, hyperbilirubinemia and ascites), serum sodium level, use of diuretics, body mass index (calculated as the body weight in kilograms divided by the square of the body height in meters) and renal function (serum creatinine) were also collected and evaluated. Patients with an urgency score ≥ 2 (once a week or more) were considered to have OAB. Significant nocturia was defined as voiding two times or more during the night, with each void preceded and followed by sleep (i.e., a score of nighttime frequency ≥ 2). Once patients with cirrhosis develop progressive abdominal heaviness and pressure as well as shortness of breath due to clinically apparent ascites, they were prescribed with furosemide 20 mg and spironolactone 50 mg daily.

The data are expressed as mean ± standard deviation for continuous variables or as a number and percentage for categorical variables. All comparisons of patients’ categorical characteristics and urinary symptoms were assessed using the Fisher’s exact test. Mann-Whitney U test was used to compare the means of continuous variables such as platelet, bilirubin, PT-INR and creatinine with or without urinary symptoms. A *p* value < 0.05 indicates statistical significance. All statistical analyses were performed using SPSS software version 17.0 (SPSS, Chicago, IL, USA).

## 3. Results

### Statistical Results

The mean age of the 158 patients was 64.29 ± 9.65 years, of whom 82 (51.9%) had chronic hepatitis B and 76 (48.1%) had chronic hepatitis C. There were 102 males and 56 females. The mean BMI (kg/m2) was 25.11 ± 4.35. The mean serum albumin level, sodium (Na), bilirubin, creatinine, platelet, and PT-INR of these patients were 3.85 ± 0.63 g/dL, 136.49 ± 3.84 mEq/L, 2.11 ± 5.88 mg/dL, 1.24 ± 1.35 mg/dL, 121.16 ± 64.44 × 10^3^/μL, 1.16 ± 0.19, respectively (Table 1).

In Table 2, 49 of 158 (31.01%) liver cirrhosis patients had OAB according to OABSS. Ninety eight of the 158 patients (62%) had significant nocturia symptoms (nocturia score ≥2) which were 61.77% in males and 62.50% in females. Fifty patients (31.65%) had urgency symptoms (urgency score ≥2) which were 29.41% in males and 35.71% in females. 29 patients (18.30%) had urge incontinence (urge incontinence score ≥2) which were 16.67% in males and 21.42% in females. There was no significant difference between genders in symptoms of frequency, nocturia, urgency, and urge incontinence.

Age was a dependent risk factor for urgency (urgency and non-urgency, 68.36 ± 10.59 years and 63.35 ± 8.79 years, *p* = 0.002), nocturia (nocturia and non-nocturia 67.15 ± 9.02 years and 61.32 ± 9.62 years, *p* < 0.001) and urge incontinence (urge incontinence and non-urge incontinence 69.76 ± 10.40 years and 63.85 ± 9.17 years, *p* = 0.008). A lower platelet level was noted in patients with an urgency score ≥2 (*p* = 0.025). Patients with nocturia and urge incontinence also had significantly lower serum albumin levels (*p* = 0.028 and 0.044, respectively). The international normalized ratio (INR) of prothrombin time was relatively longer in the patients with nocturia than in those without (*p* = 0.01) (Table 3).

## 4. Discussion

Our study found that a high proportion of patients with liver cirrhosis have OAB. Many medical disorders can cause OAB, such as cerebrovascular disease, cardiovascular disease, hypertension, diabetes mellitus, sleep disorders and metabolic syndrome [11,12,13,14]. Our research found that the prevalence of OAB in patients with liver cirrhosis was 31.01%, with a prevalence rate of urge incontinence of 18.30%, which is higher than in the age-matched general population of Taiwan. If some interference factors such as DM and hypertension are ruled out, the prevalence of OAB in patients with liver cirrhosis was 32.30%, with a prevalence rate of urge incontinence of 19.30%. The differences between countries and race could be related to a variety of factors, such as cultural differences, lifestyle preferences (factors such as obesity and caffeine consumption), and ethnicity (some nationalities may be genetically more susceptible to OAB than others). In our study, the proportion of OAB present in patients with liver cirrhosis is higher than that of the general population. One explanation relates to fluid redistribution which may result from portal hypertension, ascites and hypoalbuminemia. If these patients have concurrent leg edema or ascites, they could be taking diuretics which can cause polyuria, frequent urination or nocturia due to some postural changes.

In our research, we found that age is an independent factor for urgency, nocturia, and urge incontinence. The prevalence of nocturia (score ≥ 2) was 62% in patients with liver cirrhosis with an average age of 64.29 years. In the BACH (Boston Area Community Health) survey, aging was a risk factor for nocturia, with a prevalence rate of 41.2% in patients aged 60 to 79 years. A prior study revealed a similar finding of markedly increasing prevalence of OAB in men and women with advanced age [15]. Regardless of health status, circulatory dynamics, organ function, and immune competency tend to decline with aging, resulting in a potentiation of OAB symptoms.

In our study, hypoalbuminemia was found to be associated with nocturia and urinary incontinence. The average serum albumin level was 3.85 ± 0.63 g/dL in our study, which is lower than that of the general population. A recent study reported that the functional capacity of albumin in patients with cirrhosis is impaired [16]. Liver cirrhosis may cause hypoalbuminemia because albumin is synthesized exclusively in the liver. Serum albumin levels fall as the synthetic function of the liver declines with worsening cirrhosis. Thus, serum albumin levels can be used to gauge the severity of cirrhosis. In healthy individuals, human serum albumin constitutes 50% of the plasma proteins. The functional characteristics of albumin include plasma oncotic pressure, solubility, transport and metabolism, antioxidants, immunomodulation, capillary permeability, hemostatic effects and endothelial stabilization [17,18]. A low serum albumin level will cause a decrease in osmotic pressure in the body, triggering the onset of compensatory diuresis in order to retain fluid homeostasis and restoration of osmotic pressure [19]. Besides, a low serum albumin level, coupled with impaired albumin function, may lead to fluid accumulation in the dependent parts of the body, from which fluids can then redistribute to the circulating volume in the case of recumbent position, resulting in nocturia. The other reason is that a low serum albumin level could be viewed as an indicator of impairment in hepatic protein synthesis, which also impacts the synthesis of cholinesterase, an enzyme involved in acetylcholine hydrolysis. This condition has been reported to result in delayed removal of the large acetylcholine burst secreted by the parasympathetic nerve terminals in the bladder when the micturition reflex is activated [20]. Sugaya et al. also reported a significant association between the serum levels of cholinesterase and albumin [21]. The presence of a cholinergic effect will cause a bladder spasm, resulting in frequency and urgency.

Poor liver function may lead to OAB. Prothrombin INR abnormal also reflects hepatic synthesis dysfunction. Levels of procoagulant proteins, such as prothrombin, factors II, V, VII and X, are decreased in patients with hepatic fibrosis and cirrhosis [22]. Some studies reported that poor liver function increases the risk of insulin resistance. An accumulating body of research has shown that diabetes and insulin resistance have a strong association with OAB. Hammarsten was the first to report that patients with faster growing prostates had higher insulin [23]. Uzun et al. reported that women with OAB have an increased risk for metabolic syndrome and insulin resistance [24,25]. We found that longer INR, which represents poor liver function, may relate to nocturia (*p* = 0.01).

Patients with OAB did have a relatively low platelet count. Thrombocytopenia has been reported to represent the more severe cases of liver cirrhosis [26]. In patients with cirrhosis, the hemostatic imbalance may tip to bleeding or thrombosis [27]. Therefore, although patients may suffer from spontaneous bleeding episodes [28], cirrhosis is also associated with hypercoagulation, and patients are at a higher risk for venous thromboembolism, portal vein thrombosis, and pulmonary embolism [27]. Bladder ischemia and hypoxia caused by hypercoagulation may be important factors leading to OAB [29]. Thus, a lower platelet count may be a predictor of the severity of urgency in patients with liver cirrhosis.

The possible pathophysiology of OAB is the involvement of a chronic inflammatory process in the bladder [30,31]. Progression of liver cirrhosis is caused by dysregulation of the balance mechanism governing immune system activation/homeostasis, which triggers a continuous inflammatory process and is mediated by secretion of various cytokines capable of causing liver fibrosis and cell death. Presence of hepatic and systemic injury is associated with a high production of pro-inflammatory cytokines such as Interleukin(IL)-1β, Tumor Necrosis Factor (TNF)-α, IL-6, IL-17, as well as anti-inflammatory cytokines, IL-10 and transforming growth factor (TGF)-β [32]. Many organs exhibit physiological changes due to inflammation, and recent studies have suggested that chronic inflammation plays a role in the pathophysiology of OAB syndrome. OAB could also be a subtype of neurogenic inflammation. [33] A decreasing concentration of serum albumin and dysfunction of albumin in cirrhotic patients will impair the functioning of antioxidants and immunomodulation [17,18], which may then exacerbate the inflammatory process of urinary bladder.

This is the first clinical study on the association between viral hepatitis-related liver cirrhosis and OAB. However, there are several limitations to this study. First, this study was performed at a single institute, and further large-scale studies are needed to support our finding. Second, we did not include an age-matched control group. Third, we only collect OABSS in these patients and the voiding diary, uroflowmery, and other urodynamic studies of them were lacking, so the voiding dysfunction of patients cannot be evaluated. [34] Third, selection bias may have been present as all patients were enrolled from our outpatient department and thus may present with liver cirrhosis of relatively minor severity. Nonetheless, LUTS-related quality of life is still an important consideration in patients with a Child-Pugh grade of A and B. For patients with a Child-Pugh grade of C, achieving control of their medical condition remains the priority. Further clinical research is needed to confirm our findings.

## 5. Conclusions

There is a high prevalence of OAB in patients with liver cirrhosis. Low serum albumin level, low platelet and longer PT-INR, considered as reliable surrogates of hepatic synthesis dysfunction in cirrhotic patients, were shown to be associated with OAB and nocturia, regardless of diuretic treatment. These findings serve to raise urologists’ and gastroenterologists’ awareness of the co-existing disease dynamics between advanced cirrhosis and OAB syndrome by identifying LFT parameters of clinical importance and predictive value. An insight into this comorbid association by the multidisciplinary team will potentially drive a timely evaluation and intervention to improve LUTS-related QoL in this vulnerable patient sub-population.

## Figures and Tables

**Table 1:** Patient characteristics.

	Type	Number
Disease		
	CHB + LC	32 (20.2%)
	CHB + LC + HCC	50 (31.64%)
	CHC + LC	15 (9.49%)
	CHC + LC + HCC	61 (38.60%)
Child-Pugh classification		
	A (score5–6)	122 (77.2%)
	B (score7–9)	29 (18.35%)
	C (score10–15)	7 (4.43%)
Gender		
	Male	102 (64.56%)
	Female	56 (35.44%)
		Mean ± standard
Age (years)		64.29 ± 9.65
BMI (kg/m^2^)		25.11 ± 4.35
Albumin (g/dL)		3.85 ± 0.63
Na(Sodium) (mEq/L)		136.49 ± 3.84
Bilirubin (mg/dL)		2.11 ± 5.88
Creatinine (mg/dL)		1.24 ± 1.35
Platelet (×10^3^/μL)		121.16 ± 64.44
PT-INR		1.16 ± 0.19

CHB: chronic hepatitis B, LC: liver cirrhosis, HCC: hepatocellular carcinoma, CHC: chronic hepatitis C, BMI: Body Mass Index, PT-INR: Prothrombin time international normalized ratio.

**Table 2:** Results of the OABSS questionnaire between genders.

Variable	Score	Total (*n* = 158)	Men (*n* = 102)	Women (*n* = 56)	*P* Value
Frequency					0.4052
	1	57 (36.08%)	38 (37.25%)	19 (33.93%)	
	2	10 (6.33%)	3 (2.94%)	7 (12.50%)	
Nocturia					0.1521
	1	48 (30.38%)	30 (29.41%)	18 (32.14%)	
	2	45 (28.48%)	24 (23.53%)	21 (37.50%)	
	3	53 (33.54%)	39 (38.24%)	14 (25.00%)	
Urgency					0.5758
	1	20 (12.66%)	11 (10.78%)	9 (16.07%)	
	2	16 (10.13%)	9 (8.82%)	7 (12.50%)	
	3	12 (7.59%)	7 (6.86%)	5 (8.93%)	
	4	15 (9.49%)	9 (8.82%)	6 (10.71%)	
	5	7 (4.43%)	5 (4.90%)	2 (3.57%)	
Urge incontinence					0.4040
	1	21 (13.29%)	10 (9.80%)	11 (19.64%)	
	2	12 (7.59%)	6 (5.88%)	6 (10.71%)	
	3	7 (4.43%)	4 (3.92%)	3 (5.36%)	
	4	9 (5.70%)	6 (5.88%)	3 (5.36%)	
	5	1 (0.63%)	1 (0.98%)	0 (0.00%)	
Overactive bladder		49 (31.01%)	30 (29.41%)	19 (33.93%)	0.8891

**Table 3:** Risk factors for urgency, nocturia and urge incontinence in the patients with liver cirrhosis.

	Urgency (*n* = 108)	Non-Urgency (*n* = 50)	*P* Value	Nocturia (*n* = 98)	Non- Nocturia (*n* = 60)	*P* Value	UI (*n* = 29)	Non-UI (*n* = 129)	*P* Value
Age	68.36 ± 10.59	63.35 ± 8.79	0.002	67.15 ± 9.02	61.32 ± 9.62	<0.001	69.76 ± 10.40	63.85 ± 9.17	0.008
BMI	26.26 ± 5.30	24.61 ± 3.81	0.084	25.45 ± 4.46	24.92 ± 4.31	0.521	26.28 ± 5.72	24.85 ± 3.99	0.153
Albumin	3.77 ± 0.67	3.88 ± 0.62	0.322	3.76 ± 0.62	3.99 ± 0.63	0.028	3.63 ± 0.61	3.89 ± 0.63	0.044
Sodium	134.37 ± 2.39	137.48 ± 3.30	0.143	135.83± 2.36	137. 62± 2.58	0.382	137.55 ± 3.09	136.96 ± 3.16	0.376
Bilirubin	2.03 ± 5.95	2.14 ± 5.88	0.912	2.20± 6.17	1.97 ± 5.43	0.812	2.31 ± 6.48	1.18 ± 0.76	0.351
Cr	1.15 ± 0.95	1.27 ± 1.51	0.594	1.36 ± 1.51	1.03 ± 1.03	0.129	1.03 ± 0.54	1.28 ± 1.48	0.370
Platelet	105.78 ± 52.79	128.50 ± 68.50	0.025	119.45 ± 64.07	124.69± 65.98	0.651	124.74 ± 66.9	105.93 ± 51.5	0.158
PT-INR	1.16 ± 0.13	1.16 ± 0.21	0.859	1.19 ± 0.22	1.11 ± 0.13	0.01	1.15 ± 0.14	1.16 ± 0.20	0.819
Gender			0.415			0.927			0.460
Male	30 (58.57%)	72 (69.32%)		63 (64.29%)	39 (65.00%)		17 (16.67%)	85 (83.33%)	
Female	20 (41.43%)	36 (30.68%)		35 (35.71%)	21 (35.00%)		12 (21.43%)	44 (78.57%)	
Ascites			0.610			0.016			0.643
yes	40 (80%)	90 (83.33%)		75 (78.57%)	55 (91.67%)		107 (82.31%)	23 (79.31%)	
no	10 (20%)	18 (16.67%)		23 (23.47%)	5 (8.33%)		22 (16.92%)	6 (20.69%)	
Diuretics			0.805			0.064			0.602
yes	42 (84%)	89 (82.40%)		77 (78.57%)	54 (90.00%)		106 (80.82%)	25 (19.08%)	
no	8 (16%)	19 (17.59%)		21 (21.43%)	6 (10.00%)		23 (85.19%)	4 (14.81%)	
Diabetes mellitus			0.710			*P* = 0.638			0.277
yes	39 (78%)	87 (80.56%)		77 (78.57%)	49 (81.67%)		105 (%)	21(%)	
no	11 (22%)	21 (19.44%)		21 (21.43%)	11 (18.33%)		24 (%)	8(%)	
Hypertension			0.617			*P* = 0.469			0.742
yes	38 (76%)	78 (72.22%)		70 (71.43%)	46 (76.67%)		94 (%)	22 (%)	
no	12 (24%)	30 (27.78%)		28 (28.57%)	14 (23.33%)		35 (%)	7 (%)	

BMI: Body Mass Index, Cr: creatinine, PT-INR: Prothrombin time international normalized ratio, UI: urge incontinence.

## Data Availability

Data are available from the Research Ethics Committee at China Medical University and Hospital (IRB Number: CMUH102-REC2-038) and China Medical University Hospital (DMR-104-044) for researchers who meet the criteria for access to confidential data. The study was designed prospectively and written informed consent was obtained from patients for both the provided treatment as well as for the use of their data in this specific study. China Medical University and Hospital Research Ethics Committee, http://web.cmuh.cmu.edu.tw/2007/IRB/index.html (accessed on 28 June 2013), Address: China Medical University Hospital, 2, Yude Road Taichung, Taiwan, Phone: +886-4-22052121#1923~1927, 1929, Fax: +886-4-22071478, Email: irb@mail.cmuh.org.tw.
